# Native nephrectomy in polycystic kidney disease patients on transplant lists: how and when?

**DOI:** 10.1007/s40620-024-01899-7

**Published:** 2024-03-21

**Authors:** Sidar Copur, Lasin Ozbek, Mustafa Guldan, Ahmet Umur Topcu, Mehmet Kanbay

**Affiliations:** 1 Department of Medicine, Koc University School of Medicine, Istanbul, Turkey; 2 Division of Nephrology, Department of Medicine, Koc University School of Medicine, 34010 Istanbul, Turkey

**Keywords:** Kidney transplantation, Autosomal dominant polycystic kidney disease, Nephrectomy, Graft survival

## Abstract

Autosomal dominant polycystic kidney disease (ADPKD), the most common hereditary kidney disease, accounts for approximately 10% of the patients on kidney transplantation waitlists. High rates of complications including hemorrhage, infections, nephrolithiasis and kidney size-related compressive complaints have been reported among ADPKD patients. Therefore, the need for routine native nephrectomy and timing of such procedure in ADPKD patients being prepared for transplantation are debated. Even though pre-transplant nephrectomy has the potential to provide fewer infectious complications due to lack of immunosuppressive medication use, such procedure has been associated with longer hospital stay, loss of residual kidney function and need for dialysis. Although simultaneous nephrectomy and transplantation could potentially lead to longer perioperative duration, perioperative complications and need for blood transfusions, this was not confirmed in cohort studies. Therefore, some institutions routinely perform simultaneous unilateral nephrectomy and kidney transplantation. In this narrative review, our aim is to evaluate the current evidence regarding the need and timing of nephrectomy in ADPKD patients in relation to kidney transplantation.

**Graphical abstract:**

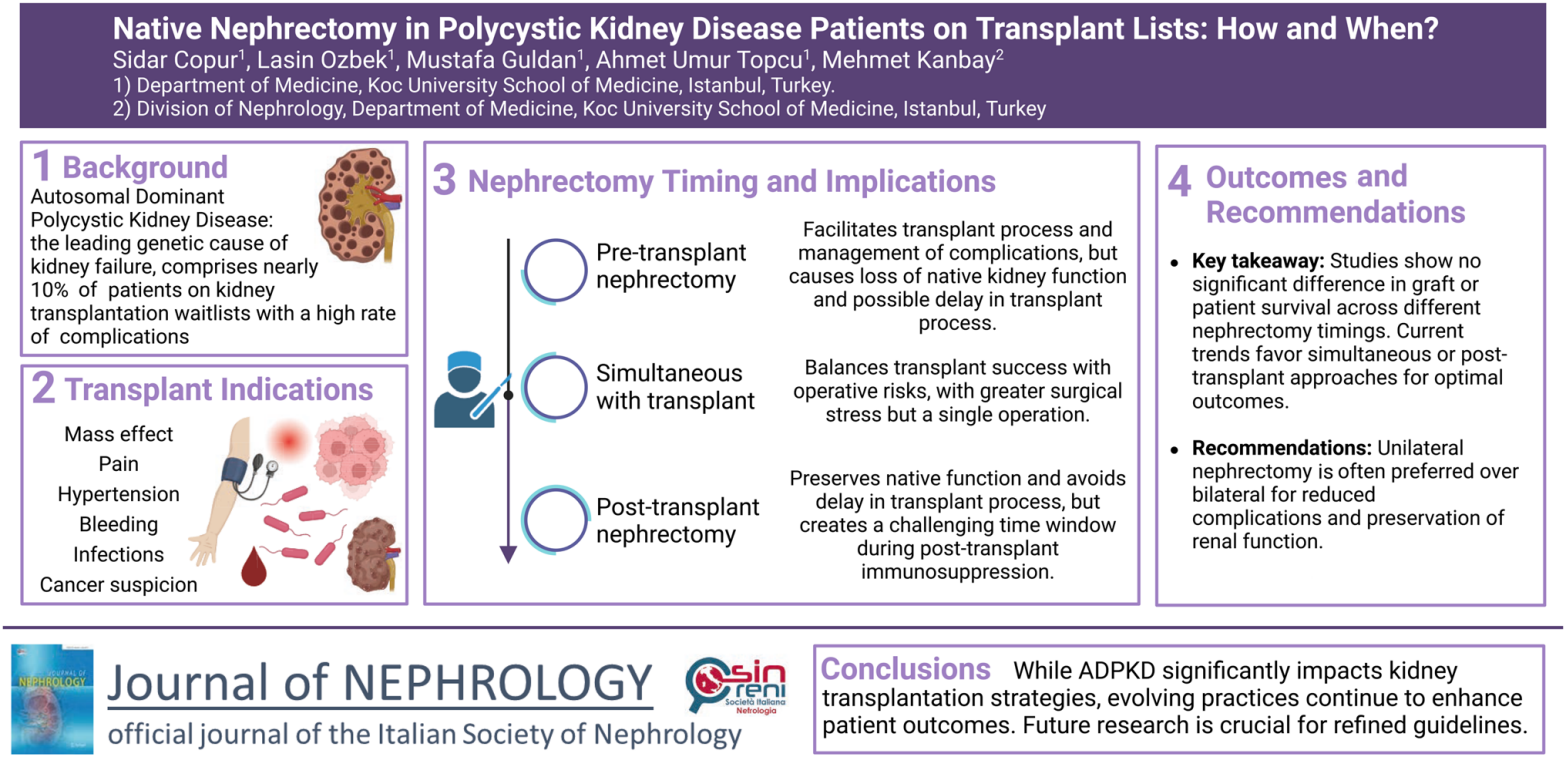

## Introduction

Autosomal dominant polycystic kidney disease (ADPKD), the most common monogenic cause of end-stage kidney disease and most common hereditary kidney disease, is caused by mutations in the polycystin-1 (PKD1) or polycystin-2 (PKD2) genes, leading to the formation of fluid-filled cysts mostly originating from distal tubules, due to abnormal cellular proliferation, fluid secretion and production of extracellular matrix [1, 2]. The prevalence of ADPKD is estimated to be between 1:400 to 1:1000 live births, while patients with ADPKD make up approximately 10% of all patients on kidney transplantation lists [3, 4]. Total kidney volume is an indicator of disease burden in patients with ADPKD and correlates with pain, hypertension, hematuria, renal function and proteinuria, thus total kidney volume is utilized as a tool to identify ADPKD patients at risk for progression to end-stage kidney disease [5, 6]. Ultrasonography, computed tomography and magnetic resonance imaging are all valid options for the assessment of total kidney volume, along with the diagnosis of common renal complications of ADPKD, including cyst infections and hemorrhage or nephrolithiasis [6]. Conventional therapeutic options  include restriction of sodium intake, increase of fluid intake and blood pressure management and are recommended for all patients, while high-risk patients with estimated glomerular filtration rate (eGFR) ≥ 25 ml/min/1.73m2 may be prescribed tolvaptan, a vasopressin V2 receptor antagonist [2]. Nevertheless, most patients (up to 70% by the age of 70) progress to kidney failure. In this relatively young population, pre-emptive living kidney transplantation is probably the best management option [7]. Native nephrectomy prior to, simultaneously with, or after kidney transplantation is occasionally performed, and indications include: [1] recurrent and/or severe cyst infections; [2] intractable pain unresponsive to analgesic medications; [3] diagnosis or suspicion of renal cell carcinoma; [4] symptomatic nephrolithiasis; [5] anatomical space considerations for transplantation; [6] recurrent and/or severe bleeding episodes [6]. In this narrative review, our aim is to evaluate the current evidence regarding the timing of nephrectomy in ADPKD patients in relation to kidney transplantation.

## Nephrectomy for ADPKD

Nephrectomy is limited to certain indications in ADPKD patients, although prevalence of such indications vary among studies. A single-center retrospective observational study including 115 ADPKD patients undergoing kidney transplantation, 68 of whom required native nephrectomy (59%), illustrated the most common indications for native nephrectomy, including infections (36%), pain (27%), hematuria (12%), suspicion of malignancy (4%), anatomical space considerations for transplantation (15%), and gastrointestinal or respiratory reasons (1%) [8]. On the other hand, indications differ considerably with regard to anatomical space considerations for transplantation (59%), recurrent cyst infections (36%), hematuria (15%), pain (24%) and suspicion of malignancy (3%). Another important aspect is the timing of the procedure with regard to kidney transplantation. Even though nephrectomy is not indicated in most cases of ADPKD, observational cohort studies illustrate high rates of nephrectomy after transplantation, i.e., up to 40% of recipients [9]. The risk of septic complications as a result of cyst infections following kidney transplantation should not be overlooked. An observational cohort study involving 99 ADPKD patients undergoing kidney transplantation, among whom 25 underwent unilateral (*n* = 19) or bilateral (*n* = 6) nephrectomy prior to transplantation and 10 had native nephrectomy after transplantation, showed that pre-transplant nephrectomy is associated with higher one- and five-year patient (100% vs. 92%; 100% vs. 84%) and graft survival rates (100% vs. 89%; 93% vs. 74%, *p* < 0.05), while cyst-related urinary tract infections appear to be the primary etiology [10]. Another retrospective observational study conducted on 73 ADPKD patients undergoing kidney transplantation illustrated higher rates of post-operative complications (34% vs. 20%), although not reaching statistical significance, in patients undergoing transplantation without nephrectomy (*n* = 43) compared to patients with pre-transplant nephrectomy (*n* = 30), while most of the complications were related to cyst infections including three cases of lethal sepsis [11]. Similar findings have been confirmed in other observational studies [12–15].

## Timing of nephrectomy

The timing of nephrectomy is an area of ongoing debate and research. Even though the polycystic kidney may expose individuals to infectious and bleeding complications, bilateral nephrectomy prior to kidney transplantation may have deleterious effects including loss of endogenous erythropoietin production, metabolism of various hormones and metabolites, and decline in quality of life with the loss of diuresis [10, 16]. Currently, there is no consensus on when or if native nephrectomy should be performed in ADPKD patients being prepared for kidney transplantation [17]. Characteristics of the studies investigating the timing of native nephrectomy in ADPKD patients undergoing kidney transplantation are summarized in Table 1.

**Table 1:** General characteristics of the studies investigating the role of native nephrectomy in autosomal dominant polycystic kidney disease patients with kidney transplantation

Study	Study Type	Total Number of Patients	Patient Groups	Age in years (Mean ± SD)	Sex (F:M or %F*)	Donor Type (Cadaver: Living or %L*)	Outcome
Brazda et al. 1996 [10]	Retrospective cohort	99 (96 adult, 3 pediatric)	Pre-transplant nephrectomy (*n* = 25)	NA	NA	NA	Pre-transplant group had better one- and five-year graft survival rates compared to the non-nephrectomy group. Graft survival outcome was better in patients without any cyst-related complications
			Post-transplant nephrectomy (*n* = 64)				
			Transplant only (*n* = 10)				
Veroux et al. 2016 [27]	Retrospective cohort	145	Pre-transplant nephrectomy (*n* = 25)	44 ± 8	10:15 (40%)	20:5 (20%)	Graft and patient survival rates at 1 and 5 years, and perioperative surgical complications were not significantly different between the groups. Yhe pre-transplant group had significantly longer pre-transplant dialysis and waiting time for transplant, and lower levels of hemoglobin and residual diuresis volumes at the time of transplant
			Simultaneous unilateral nephrectomy (*n* = 40)	51 ± 9.7	14:26 (35%)	35:5 (13%)	
			Transplant only (*n* = 80)	52 ± 10	30:50 (38%)	72:8 (10%)	
Jänigen et al. 2020 [21]	Retrospective consecutive cases	193	ADPKD patients with simultaneous ipsilateral nephrectomy (*n* =)	54.6 (95% CI 53.0–56.2)	88:105 (45.6%)	96:97 (50.3%)	The ADPKD group had higher frequency of intraoperative blood transfusions and early post-op UTI. One-year patient survival and death-censored graft survival showed no significant difference
			Matched control group without ADPKD (*n* =)	55.7 (95% CI 53.0–59.1)	61:132 (31.6%)	99:94 (51.3%)	
Rasmussen et al. 2022 [29]	Retrospective cohort	28	Pre-transplant nephrectomy (*n* = 10)	57 (8.4)	NR	10:0 (0%)	Perioperative morbidity and graft function did not differ between the groups. Total cost was lower in the simultaneous group
			Simultaneous nephrectomy (*n* = 18)	56 (8.7)	NR	3:15 (83%)	
Grodstein et al. 2017 [31]	Retrospective cohort	594	Pre-transplant bilateral nephrectomy (*n* = 27)	49.9	55.6%	37%	No significant difference in the rate of post-operative complications and delayed graft function. Compared to the transplant-only group, pre-transplant patients had more wound complications, and the simultaneous group had a lower incidence of lymphocele but higher rate of renal vascular thrombosis. Post-transplant graft survival was not significantly different between the groups
			Simultaneous bilateral nephrectomy (*n* = 161)	50.6	37.9%	59%	
			Transplant only (*n* = 303)	53.8	49.8%	38%	
Rozanski et al. 2005 [11]	Retrospective cohort	73	Pre-transplant unilateral nephrectomy (*n* = 30)	48.16	9:21 (30%)	30:0 (0%)	Graft and patient outcomes, and post-transplant complications were similar regardless of nephrectomy status
			Transplant only (*n* = 43)	49.46	22:21 (51%)	43:0 (0%)	
Mendez et al. 1975 [14]	Retrospective cohort	17	Pre-transplant bilateral nephrectomy (*n* = 8)	NR (Range, 20–55)	9:8 (53%)	17:0 (0%)	Higher mortality in transplant only group
			Transplant only (*n* = 9)				
Ho-Hsieh et al. 1987 [12]	Retrospective cohort	51	Simultaneous nephrectomy (*n* = 24)	47.5	20:31 (39%)	49:7 (13%)	Graft success at one year was 78% vs 58% in patients with and without pre-transplant bilateral nephrectomy, respectively (not significant.)
			Transplant only (*n* = 27)				
Rayner et al. 1990 [13]	Retrospective cohort	19	Simultaneous bilateral nephrectomy (*n* = 6)	NA	NA	NA	Increased mortality and morbidity caused by complications related to polycystic kidney disease in transplant only group
			Transplant only (*n* = 13)				
Neeff et al. 2013 [18]	Retrospective consecutive cases	100	Simultaneous unilateral nephrectomy	54.7	41:59 (41%)	62:38 (38%)	No comparison in terms of patient or graft survival rates due to lack of any control group
Lucas et al. 2010 [19]	Retrospective cohort	38	Simultaneous unilateral nephrectomy (*n* = 16)	53.5 (median)	8:8 (50%)	6:10 (40%)	No significant morbidity difference between groups
			Transplant only (*n* = 22)	48.3 (median)	10:12 (%45)	15:6 (1 is unknown)	
Nunes et al. 2007 [20]	Retrospective cohort	159	Simultaneous nephrectomy (*n* = 16)	49.38	33.3%	6.2%	No differences in rates of delayed graft function, acute rejection, chronic allograft dysfunction, graft function at 1 month as well as at 1 and 5 years, and patient and graft survival at 1 and 5 years
			No nephrectomy (*n* = 143)	50.10	47%	2.2%	
Tabibi et al. 2005[22]	Retrospective cohort	33	Simultaneous nephrectomy (*n* = 13)	37.7 (range, 28 to 50)	NR	NR	No difference in morbidity
			No nephrectomy (*n* = 20)	45.4 (range, 33 to 58)			
Fuller et al. 2005 [23]	Retrospective cohort	32	Pre-transplant unilateral nephrectomy (*n* = 7)	41.8	4:3 (%57)	NA	No differences in perioperative morbidity and post-operative outcome when comparing pre-transplant, concomitant and post-transplant native nephrectomy
			Simultaneous nephrectomy (*n* = 16)	37.4	7:9 (44%)	2:14 (88%)	
			Post-transplant nephrectomy (*n* = 9)	49.4	5:4 (56%)	5:4 (44%)	
Drognitz et al. 2006 [24]	Retrospective cohort	49	Simultaneous unilateral Nephrectomy (*n* = 43)	NA***	NA***	NA***	Patient survival, graft function and complications did not differ between the groups. ***Outcomes not specific for ADPKD patients but for all
			No nephrectomy (*n* = 2)				
Kim et al. 2016 [25]	Retrospective cohort	41	Simultaneous nephrectomy (*n* = 13)	46.2 ± 8.3	7:6 (54%)	4:9 (69%)	No difference in graft survival, early and late complications
			Previous nephrectomyor without nephrectomy (*n* = 28)	48.2 ± 7.9	10:18 (36%)	6:22 (79%)	
Kirkman et al. 2011 [26]	Retrospective cohort	35	Pre-transplant nephrectomy (*n* = 20)	51.5 (median), (range, 43–65)	19:16 (54%)	NR	Pre-transplant group had higher morbidity, the safest approach was found to be post-transplant unilateral nephrectomy
			Post-transplant nephrectomy (*n* = 12)				
			Sandwich technique (*n* = 3)				
Abrol et al. 2021 [32]	Retrospective case–control	148	Simultaneous bilateral nephrectomy (*n* = 51)	52.7 ± 10.1 (range, 29.2–77.7)	22:29, (43%)	0:51 (100%)	No difference in kidney function and rate of delayed graft function. Simultaneous nephrectomy and transplant patients required longer ICU stay, higher rates of blood transfusions, longer hospital stays and had longer cold ischemia time
			No nephrectomy (*n* = 97)	55.3 ± 10.9 (range, 26.3–75.9)	45:52 (46%)	0:97 (100%)	
Glassman et al. 2000 [33]	Retrospective cohort	23	Pre-transplant bilateral nephrectomy (*n* = 4)	Groups are age-matched (no further details)	Groups are sex-matched (no further details)	NR	No difference in terms of mortality and morbidity between the groups
			Simultaneous bilateral nephrectomy (*n* = 10)				
			No nephrectomy (*n* = 9)				
Wagner et al. 2007 [34]	Retrospective cohort	68 (only patients with living donor transplantation were further scrutinized, *n* = 39)	Pre-transplant nephrectomy (*n* = 15)	51 ± 9.5	6:9 (40%)	29:39 (57%)	No difference between patient survival and graft function between simultaneous or staged groups
			Simultaneous bilateral nephrectomy (*n* = 17)	54 ± 6.8	9:8 (53%)		
			No nephrectomy (*n* = 7)	NR	NR		
Kramer et al. 2009 [35]	Retrospective consecutive cases	20	Simultaneous nephrectomy (No comparison group)	42 (range, 32- 63)	7:13 (35%)	0:20 (%100)	Graft and patient survival following the combined procedure was 100%
Casteleijn et al. 2023 [39]	Retrospective cohort	391	Pre-transplant nephrectomy (*n* = 114)	54 ± 8	43:71 (38%)	202:189 (48%)	No difference in 10-year patient survival and 10-year death-censored graft survival
			Post-transplant nephrectomy (*n* = 20)	53 ± 7	3:17 (15%)		
			No nephrectomy (*n* = 257)	54 ± 10	129:129 (50%)		
Patel et al. 2011 [40]	Retrospective cohort	157 (only patients with native nephrectomy were scrutinized, *n* = 31)	Pre-transplant nephrectomy (*n* = 10)	49	12:19 (%61)	114:43 (%27)	No difference in the rate of complications between pre- and post-transplant groups. Post-transplant patients had more severe complications
			Simultaneous nephrectomy (*n* = 1)				
			Post-transplant (*n* = 20)				
García-Rubio et al. 2015 [38]	Retrospective cohort	87	Pre-transplant nephrectomy (*n* = 27)	53.3	13:15 (%46)	0%	Graft survival rates at one and five years, and post-operative complications showed no difference
			Transplant only (*n* = 60)	56.03	44:14 (%29)		
Maxeiner et al. 2019 [41]	Retrospective cohort	121	Pre-transplant nephrectomy (*n* = 89)	53.92	%30.3	NR	Higher rates of serious post-operative complications and longer hospital stay in pre-transplant group but no effect on graft function
			Post-transplant nephrectomy (*n* = 32)	53.75	%31.2		

*CI* confidence interval; *F* female; *M* male; *NA* not applicable; *NR* not reported; *SD* standard deviation

### Simultaneous approach

Unilateral native nephrectomy with simultaneous kidney transplantation is a surgical approach aiming to minimize complications such as loss of physiological function of the native kidney along with infectious, bleeding or malignant complications.

A single-center observational cohort study involving 100 simultaneous ipsilateral nephrectomies with kidney transplantation demonstrated that such surgical approach is safe and effective and achieved 97% one-year patient survival and 96% one-year graft survival rates. Moreover, 95% patient survival and 80% graft survival rates were achieved along with a mean serum creatinine level of 1.49 (range 0.8–2.8) mg/dL at the five-year follow-up visit. The rate of surgical complications requiring re-operation was attributable to unilateral nephrectomy in 12% (lymphocele 4%, hernia 4%, post-operative hematoma or bleeding 4%). The surgical procedure for nephrectomy in this study included an extra-peritoneal curvilinear incision in the lower abdominal quadrant (Gibson incision), and only 38 patients received kidney allografts from living donors [18]. This study clearly indicates the safety of kidney transplantation with unilateral nephrectomy, reporting high graft and patient survival and low rates of surgical complications, at least in experienced hands.

Another retrospective cohort study conducted on 42 ADPKD patients aimed to compare the efficiency and safety of ipsilateral nephrectomy with transplantation (*n* = 16) to transplantation alone (*n* = 22) and unilateral (*n* = 18) to bilateral laparoscopic native nephrectomy (*n* = 24). No statistically significant difference was reported between ipsilateral nephrectomy with transplantation to transplantation alone groups in terms of operative time (236 vs. 208 min, *p* = 0.104), estimated blood loss (250 vs. 200 ml, *p* = 0.37), serum creatinine at discharge (1.50 vs. 1.60 mg/dl, *p* = 0.49) or serious post-operative complications. On the other hand, bilateral laparoscopic nephrectomy required greater operative time (270 vs. 180 min, *p* < 0.001) and estimated blood loss (125 vs. 50 ml, *p* < 0.001), without difference in post-operative serum creatinine level (1.20 vs. 1.15 mg/dl, *p* = 0.55) or median hospital stay compared to unilateral laparoscopic nephrectomy [19].

A further retrospective cohort study involving 159 ADPKD patients undergoing kidney transplantation evaluated the surgical and medical outcomes of patients requiring simultaneous unilateral native nephrectomy (*n* = 143) or not (*n* = 16). Patients requiring unilateral native nephrectomy showed longer surgical time (4.23 vs. 3.01 h, *p* < 0.001), higher crystalloid infusions (2.76 vs. 1.84 L, *p* < 0.001) and blood transfusions (2.93 vs. 2.07 units, *p* < 0.05), however, there was no difference in terms of hospital stay (16.5 vs. 12.7 days). No statistically significant difference was reported in terms of delayed graft function (12.5% vs. 19.9%), acute rejection (33.3% vs. 25.5%) or chronic allograft dysfunction (28.6 vs. 15.8%). Serum creatinine measurements were similar at one-month (1.60 vs. 1.79 mg/dl), one-year (1.39 vs. 1.38 mg/dl) and five-year follow-up visits (1.47 vs. 1.29 mg/dl). Moreover, no differences were detected in terms of allograft survival at one-year (93.3% vs. 91.6%) or five-year follow-up (86.4% vs. 79.4%) in patients with similar etiologies for graft loss [20]. The interest of this study resides in the comparison between two approaches for the management of ADPKD with a long follow-up period (8.53 vs. 6.36 years) and high number of patients (*n* = 159), while the limitation is the relatively low number of patients not undergoing native nephrectomy [20].

Additionally, Jänigen et al. showed that simultaneous ipsilateral nephrectomy had comparable morbidity in end-stage kidney transplant recipients with ADPKD. However, it resulted in higher blood transfusion rates (22.8% vs. 6.7%, *p* < 0.0001), prolonged surgery time (169 min vs. 139 min, *p* < 0.0001), and increased early postoperative urinary tract infections (40.4% vs. 29.0%, *p* = 0.0246) [21]. Five other retrospective observational cohort studies with small sample size reported similar outcomes with unilateral nephrectomy performed simultaneously with transplantation [22–26]. Moreover, the safety of simultaneous unilateral native nephrectomy in terms of perioperative and post-operative complications has been reported in several other cohort studies [21, 27, 28]. Furthermore, the simultaneous surgical approach has proven to be more cost-effective mostly due to the shorter hospital stay compared to the staged surgical approach [29]. On the other hand, in comparing two-staged versus simultaneous native nephrectomy and kidney transplantation in ADPKD patients, analysis of seven retrospective cohort studies (385 patients) revealed that staged procedures were linked to a significantly longer cumulative operative time (RR 1.86; *p* = 0.01) and an elevated risk of blood transfusions (RR 2.69; *p* < 0.00001) [30]. Nevertheless, there were no notable differences in hospitalization length, major complications, or vascular thromboses during the transplant procedure [30]. These findings underscore the importance of individualized decision-making for ADPKD patients undergoing kidney transplantation.

Another crucial consideration is whether unilateral or bilateral native nephrectomy should be performed. Experience on bilateral native nephrectomy is highly limited. A single-center retrospective cohort study evaluated the efficiency and safety of simultaneous bilateral native nephrectomy (*n* = 161) in comparison with either transplantation alone (*n* = 303) or pre-transplant bilateral nephrectomy (*n* = 27) in 569 ADPKD patients. Ten-year graft survival rates were 68.5% for the transplantation alone group, 63.6% for the simultaneous procedure and 65.7% for the pre-transplant nephrectomy group, with no statistically significant difference among them. No significant difference was found in terms of post-operative complications, including delayed graft function, while wound infections were more commonly encountered in the pre-transplant nephrectomy group (*p* = 0.03), and lymphocele was less likely to be detected in the simultaneous procedure group (*p* = 0.002). Nevertheless, the simultaneous procedure group showed higher rates of renal vascular thrombosis (4.4% vs 1.3% transplant alone, 0% pre; *p* = 0.04) [31]. Moreover, a cohort study involving 148 ADPKD patients receiving kidney transplantation showed that patients undergoing simultaneous bilateral native nephrectomy (*n* = 51) experienced longer cold ischemia time and longer intensive care unit and hospital stay compared to patients undergoing transplantation alone (*n* = 97). Nevertheless, surgical complications, hospital re-admissions or renal function over the one-year follow-up period were similar between the two groups [32]. Both the safety and efficiency of such surgical procedure were compared with transplantation alone and staged-approach for transplantation and nephrectomy in another retrospective cohort study involving 23 ADPKD patients. Higher operative time and intraoperative blood loss were observed in patients undergoing the simultaneous procedure compared to patients undergoing transplantation alone, but not to the staged-approach [33]. Such findings have been confirmed in other observational studies with small sample size [34, 35].

Within the field of nephrectomy, recent advances include robotic surgery. As highlighted by Masterson et al., this approach demonstrates favorable operative times and outcomes, notably contributing to increased graft survival and reduced mortality rates when compared to traditional open procedures [36].

### Pre- or post-transplant nephrectomy

The main disadvantages of pre-transplant nephrectomy compared to the post-transplant procedure include [1] longer hospital stay, most likely due to the need for dialysis; [2] lower quality of life, either due to more intense dialysis schedules or fluid intake restrictions; [3] loss of the physiological function of native kidneys including production and secretion of various hormones and cytokines, including erythropoietin. Moreover, the native kidney may remain stable or diminish in size after kidney transplantation which could reduce the need for native nephrectomy due to anatomical space-related or compression-related causes (i.e. respiratory or gastrointestinal complaints, pain) [37]. Even though these factors may advocate for a restricted pre-transplant nephrectomy approach, infectious complications can predispose to sepsis or urgent nephrectomy, possibly impairing allograft function. Few observational studies have investigated and compared such approaches, and there is a need for large-scale future studies in order to better understand this issue.

A retrospective cohort study including 87 patients with ADPKD undergoing kidney transplantation, 27 (30%) of whom underwent pre-transplant nephrectomy, showed no statistically significant difference in terms of one-year (98% vs. 95%) or five-year (95% vs. 80%) allograft survival and post-operative complications. On the other hand, serum creatinine levels at three- (1.57 vs. 2.03 mg/dl) and six-month (1.50 vs. 1.83 mg/dl) follow-up showed some differences, favoring pre-transplant nephrectomy, despite not reaching statistical significance [38].

A large-scale, retrospective, observational, single-center cohort study involving 391 ADPKD patients undergoing kidney transplantation evaluated the role of either pre- (*n* = 114) or post-transplant (*n* = 30) nephrectomy compared to no nephrectomy (*n* = 257). The most common indication for pre-transplant nephrectomy involved anatomical space considerations for the transplantation procedure (49.6%), followed by cyst infections (28.1%), cyst hemorrhage (23%) and pain (20%), while the most common indications for post-transplant nephrectomy were infectious complications (59.5%), followed by pain (24.3%) and gastrointestinal complaints (18.9%). No statistically significant difference was detected in terms of the size of the removed kidney (*p* = 0.50), type of surgical approach (open vs. laparoscopic, *p* = 0.10), or rates of surgical complications (38.3% vs. 27.0%, *p* = 0.20). However, nephrectomy performed in the post-transplant period showed shorter length of hospital stay (6.0 vs. 10.0 days, *p* < 0.001). No statistically significant difference was found between pre- and post-transplant nephrectomy in terms of delayed graft function (22.5% vs. 20.0% *p* = 0.90), graft failure (11.4% vs. 10.0%, *p* = 0.90), eGFR at follow-up (49 vs. 47 ml/min/1.73 m2, *p* = 0.80) or mortality (31.9% vs. 20.0%, *p* = 0.30) over a median follow-up of 83 months. Similarly, no difference was detected when compared to the no nephrectomy group [39].

Another retrospective observational cohort study involving 157 ADPKD patients undergoing kidney transplantation with 31 patients requiring native nephrectomy reported similar rates of surgical complications for native nephrectomy performed in the pre- (10 patients) or post-transplant (20 patients) period, however, severe complications mostly occurred in the post-transplant group. Moreover, the laparoscopic procedure has been linked to lower rates of surgical complications (20%) compared to the open procedure (73%) in a statistically significant manner, and the laparoscopic procedure has been associated with shorter hospital stay (5 vs. 12 days, *p* = 0.003) [40].

Conversely, another retrospective cohort study involving 121 ADPKD patients undergoing kidney transplantation with either pre-transplant (*n* = 89) or post-transplant (*n* = 32) nephrectomy reported contradictory findings, with higher rates of serious post-operative complications and longer hospital stay in the pre-transplant nephrectomy group. However, no effect on graft function was detected [41].

## Considerations and suggestions for the future

Prospective studies with larger cohorts and extended follow-up periods are imperative to establish more conclusive evidence regarding the timing of nephrectomy and its impact on long-term patient and graft outcomes. Comparative analyses between different surgical approaches, particularly assessing the safety of simultaneous nephrectomy with transplantation, could further guide clinicians in selecting the most appropriate interventions.

Exploring the functional outcomes of nephrectomy, including its influence on renal function, is crucial for understanding the physiological consequences of surgical interventions. Moreover, incorporating measures of quality of life will provide valuable insights into patients' experiences, helping tailor interventions to enhance overall well-being.

Given the limited clinical experience with bilateral native nephrectomy, future studies should specifically focus on evaluating the safety and efficiency of this approach, comparing outcomes with unilateral nephrectomy. Patient-centered outcomes, including pain reduction, symptom relief, and overall satisfaction, should be emphasized in future studies to provide a comprehensive understanding of the impact of nephrectomy.

Health economics analyses comparing cost-effectiveness of different nephrectomy approaches are needed for informing healthcare policies and resource allocation.

## Conclusions

**Fig. 1:**
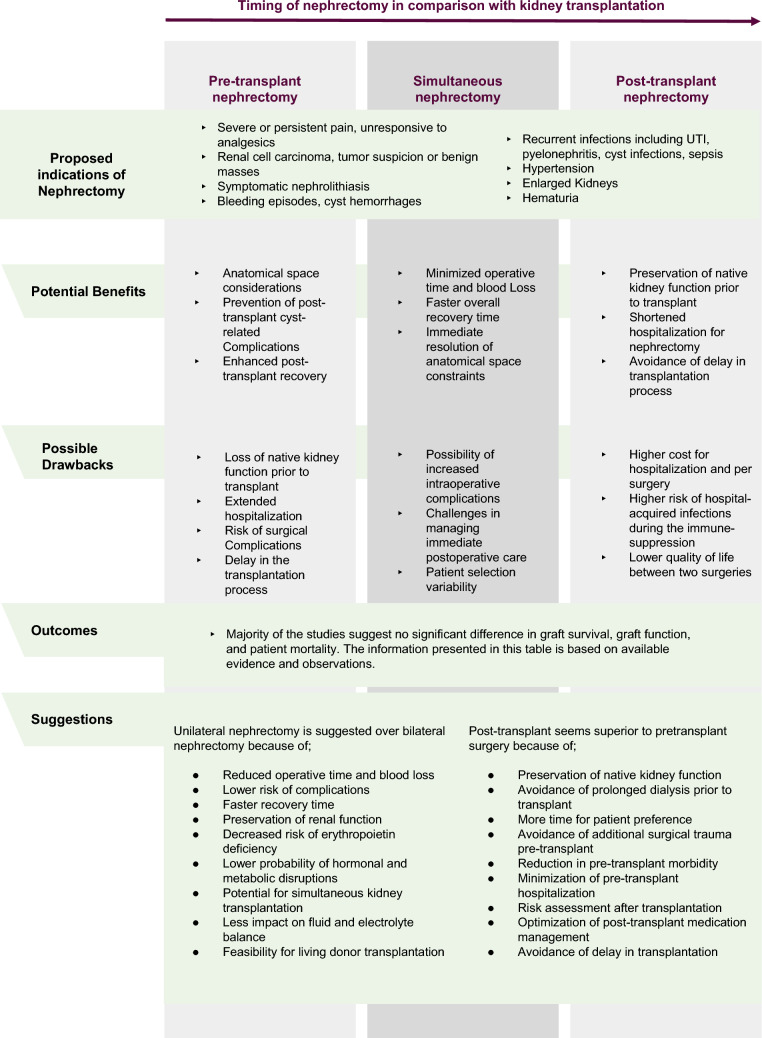
Comparison of pre-transplant, simultenous and post-transplant nephrectomy approaches for patients with autosomal dominant polycystic kidney disease

ADPKD, the most common monogenic cause of end-stage kidney disease, accounts for approximately 10% of the patients waitlisted for kidney transplantation. Currently, there is no consensus on whether or when native nephrectomy should be performed in such patients in relation to the transplantation procedure [42]. Potential advantages and disadvantages of such therapeutic options are depicted in Fig. 1. In the review of optimal timing for native nephrectomy in individuals with ADPKD, the absence of a consensus underscores the need for a patient-tailored approach. Future large-scale prospective clinical trials are needed in order to achieve a better understanding of this issue.

## References

[1] Cornec-Le Gall E, Alam A, Perrone RD (2019) Autosomal dominant polycystic kidney disease. Lancet 393(10174):919–935 30819518 10.1016/S0140-6736(18)32782-X

[2] Reiterová J, Tesař V (2022) Autosomal dominant polycystic kidney disease: from pathophysiology of cystogenesis to advances in the treatment. Int J Mol Sci 23(6):3317 35328738 10.3390/ijms23063317 PMC8949594

[3] Willey CJ, Blais JD, Hall AK, Krasa HB, Makin AJ, Czerwiec FS (2017) Prevalence of autosomal dominant polycystic kidney disease in the European union. Nephrol Dial Transplant 32(8):1356–1363 27325254 10.1093/ndt/gfw240 PMC5837385

[4] Spithoven EM, Kramer A, Meijer E, Orskov B, Wanner C, Abad JM et al (2014) Renal replacement therapy for autosomal dominant polycystic kidney disease (ADPKD) in Europe: prevalence and survival–an analysis of data from the ERA-EDTA registry. Nephrol Dial Transplant 29 Suppl 4(Suppl 4):iv15-25 25165182 10.1093/ndt/gfu017 PMC7611099

[5] Grantham JJ, Torres VE, Chapman AB, Guay-Woodford LM, Bae KT, King BF Jr et al (2006) Volume progression in polycystic kidney disease. N Engl J Med 354(20):2122–2130 16707749 10.1056/NEJMoa054341

[6] Chapman AB, Devuyst O, Eckardt KU, Gansevoort RT, Harris T, Horie S et al (2015) Autosomal-dominant polycystic kidney disease (ADPKD): executive summary from a kidney disease: improving global outcomes (KDIGO) controversies conference. Kidney Int 88(1):17–27 25786098 10.1038/ki.2015.59 PMC4913350

[7] Niemczyk M, Niemczyk S, Paczek L (2009) Autosomal dominant polycystic kidney disease and transplantation. Ann Transplant 14(4):86–90 20009161 PMC2843931

[8] Navrátil P, Špaček J, Balík M, Novák I, Pacovsky J, Navrátil St P, Guňka I (2023) Native nephrectomy in patients with autosomal dominant polycystic kidney disease in the kidney transplant program - single-center retrospective results of 2000–2020. Rozhl Chir 102(1):11–16 36809889 10.33699/PIS.2023.102.1.11-16

[9] Sulikowski T, Tejchman K, Zietek Z, Rózański J, Domański L, Kamiński M et al (2009) Experience with autosomal dominant polycystic kidney disease in patients before and after renal transplantation: a 7-year observation. Transplant Proc 41(1):177–180 19249508 10.1016/j.transproceed.2008.10.034

[10] Brazda E, Ofner D, Riedmann B, Spechtenhauser B, Margreiter R (1996) The effect of nephrectomy on the outcome of renal transplantation in patients with polycystic kidney disease. Ann Transplant 1(2):15–18 9869924

[11] Rozanski J, Kozlowska I, Myslak M, Domanski L, Sienko J, Ciechanowski K, Ostrowski M (2005) Pretransplant nephrectomy in patients with autosomal dominant polycystic kidney disease. Transplant Proc 37(2):666–668 15848495 10.1016/j.transproceed.2004.12.115

[12] Ho-Hsieh H, Novick AC, Steinmuller D, Streem SB, Buszta C, Goormastic M (1987) Renal transplantation for end-stage polycystic kidney disease. Urology 30(4):322–326 3310365 10.1016/0090-4295(87)90293-7

[13] Rayner BL, Cassidy MJ, Jacobsen JE, Pascoe MD, Pontin AR, van Zyl SR (1990) Is preliminary binephrectomy necessary in patients with autosomal dominant polycystic kidney disease undergoing renal transplantation? Clin Nephrol 34(3):122–124 2225563

[14] Mendez R, Mendez RG, Payne JE, Berne TV (1975) Renal transplantation. In adult patients with end stage polycystic kidney disease. Urology 5(1):26–27 1090045 10.1016/0090-4295(75)90295-2

[15] Wetzel O, Hormi M, Le Normand L, Karam G, Guenel J, Auvigne J, Buzelin JM (1993) Autosomal dominant polycystic kidney disease: urologic complications and results of kidney transplantation: 217 patients. Prog Urol 3(2):252–262 8508209

[16] Calman KC, Bell PR, Briggs JD, Hamilton DN, MacPherson SG, Paton AM (1976) Bilateral nephrectomy prior to renal transplantation. Br J Surg 63(7):512–516 782622 10.1002/bjs.1800630704

[17] El Chediak A, Degheili JA, Khauli RB (2021) Genitourinary interventions in autosomal dominant polycystic kidney disease: clinical recommendations for urologic and transplant surgeons. Exp Clin Transplant 19(2):95–103 33494664 10.6002/ect.2020.0292

[18] Neeff HP, Pisarski P, Tittelbach-Helmrich D, Karajanev K, Neumann HP, Hopt UT, Drognitz O (2013) One hundred consecutive kidney transplantations with simultaneous ipsilateral nephrectomy in patients with autosomal dominant polycystic kidney disease. Nephrol Dial Transplant 28(2):466–471 23042709 10.1093/ndt/gfs118

[19] Lucas SM, Mofunanya TC, Goggins WC, Sundaram CP (2010) Staged nephrectomy versus bilateral laparoscopic nephrectomy in patients with autosomal dominant polycystic kidney disease. J Urol 184(5):2054–2059 20850813 10.1016/j.juro.2010.06.150

[20] Nunes P, Mota A, Alves R, Figueiredo A, Parada B, Macário F, Rolo F (2007) Simultaneous renal transplantation and native nephrectomy in patients with autosomal-dominant polycystic kidney disease. Transplant Proc 39(8):2483–2485 17954154 10.1016/j.transproceed.2007.07.035

[21] Jänigen BM, Hempel J, Holzner P, Schneider J, Fichtner-Feigl S, Thomusch O et al (2020) Simultaneous ipsilateral nephrectomy during kidney transplantation in autosomal dominant polycystic kidney disease: a matched pair analysis of 193 consecutive cases. Langenbecks Arch Surg 405(6):833–842 32705344 10.1007/s00423-020-01939-3 PMC7471159

[22] Tabibi A, Simforoosh N, Abadpour P, Gholamrezaie HR, Nafar M (2005) Concomitant nephrectomy of massively enlarged kidneys and renal transplantation in autosomal dominant polycystic kidney disease. Transplant Proc 37(7):2939–2940 16213267 10.1016/j.transproceed.2005.07.053

[23] Fuller TF, Brennan TV, Feng S, Kang SM, Stock PG, Freise CE (2005) End stage polycystic kidney disease: indications and timing of native nephrectomy relative to kidney transplantation. J Urol 174(6):2284–2288 16280813 10.1097/01.ju.0000181208.06507.aa

[24] Drognitz O, Kirste G, Schramm I, Assmann A, Pohl M, Gobel H et al (2006) Kidney transplantation with concomitant unilateral nephrectomy: a matched-pair analysis on complications and outcome. Transplantation 81(6):874–880 16570011 10.1097/01.tp.0000203319.93328.27

[25] Kim JH, Chae SY, Bae HJ, Kim JI, Moon IS, Choi BS et al (2016) Clinical outcome of simultaneous native nephrectomy and kidney transplantation in patients with autosomal dominant polycystic kidney disease. Transplant Proc 48(3):840–843 27234748 10.1016/j.transproceed.2015.08.047

[26] Kirkman MA, van Dellen D, Mehra S, Campbell BA, Tavakoli A, Pararajasingam R et al (2011) Native nephrectomy for autosomal dominant polycystic kidney disease: before or after kidney transplantation? BJU Int 108(4):590–594 21166760 10.1111/j.1464-410X.2010.09938.x

[27] Veroux M, Zerbo D, Basile G, Gozzo C, Sinagra N, Giaquinta A et al (2016) Simultaneous native nephrectomy and kidney transplantation in patients with autosomal dominant polycystic kidney disease. PLoS ONE 11(6):e0155481 27257690 10.1371/journal.pone.0155481 PMC4892472

[28] Gadelkareem RA, Abdelgawad AM, Mohammed N (2022) Simultaneous kidney transplantation and ipsilateral native nephrectomy in patients with autosomal dominant polycystic kidney disease. World J Transplant 12(9):310–312 36187882 10.5500/wjt.v12.i9.310 PMC9516487

[29] Rasmussen A, Levine MA, Mandurah MM, Sener A, Luke PP (2022) Staged vs. simultaneous bilateral nephrectomy and kidney transplantation in patients with autosomal dominant polycystic kidney disease: outcomes and costs. Can Urol Assoc J 16(12):424–429 36656695 10.5489/cuaj.7816 PMC9851214

[30] Xu J, D’Souza K, Lau NS, Leslie S, Lee T, Yao J et al (2022) Staged versus concurrent native nephrectomy and renal transplantation in patients with autosomal dominant polycystic kidney disease: a systematic review. Transplant Rev (Orlando) 36(1):100652 34688508 10.1016/j.trre.2021.100652

[31] Grodstein EI, Baggett N, Wayne S, Leverson G, D’Alessandro AM, Fernandez LA et al (2017) An evaluation of the safety and efficacy of simultaneous bilateral nephrectomy and renal transplantation for polycystic kidney disease: a 20-year experience. Transplantation 101(11):2774–2779 29064957 10.1097/TP.0000000000001779

[32] Abrol N, Bentall A, Torres VE, Prieto M (2021) Simultaneous bilateral laparoscopic nephrectomy with kidney transplantation in patients with ESRD due to ADPKD: a single-center experience. Am J Transplant 21(4):1513–1524 32939958 10.1111/ajt.16310

[33] Glassman DT, Nipkow L, Bartlett ST, Jacobs SC (2000) Bilateral nephrectomy with concomitant renal graft transplantation for autosomal dominant polycystic kidney disease. J Urol 164(3 Pt 1):661–664 10953121 10.1097/00005392-200009010-00011

[34] Wagner MD, Prather JC, Barry JM (2007) Selective, concurrent bilateral nephrectomies at renal transplantation for autosomal dominant polycystic kidney disease. J Urol 177(6):2250–2254 17509331 10.1016/j.juro.2007.01.146

[35] Kramer A, Sausville J, Haririan A, Bartlett S, Cooper M, Phelan M (2009) Simultaneous bilateral native nephrectomy and living donor renal transplantation are successful for polycystic kidney disease: the University of Maryland experience. J Urol 181(2):724–728 19091353 10.1016/j.juro.2008.10.008

[36] Masterson JM, Zhao H, Taich L, Naser-Tavakolian A, Johnson H, Najjar R et al (2023) Robotic bilateral nephrectomy for large polycystic kidney disease. BJUI Compass 4(6):701–708 37818019 10.1002/bco2.263 PMC10560624

[37] Yamamoto T, Watarai Y, Kobayashi T, Matsuda Y, Tsujita M, Hiramitsu T et al (2012) Kidney volume changes in patients with autosomal dominant polycystic kidney disease after renal transplantation. Transplantation 93(8):794–798 22491657 10.1097/TP.0b013e318246f910

[38] García-Rubio JH, Carrasco Valiente J, Campos Hernández JP, Ruiz García J, Márquez López J, Regueiro López JC et al (2015) Graft survival in patients with polycystic kidney disease with nephrectomy of native kidney pretransplant. Transplant Proc 47(9):2615–2617 26680051 10.1016/j.transproceed.2015.10.009

[39] Casteleijn NF, Geertsema P, Koorevaar IW, Inkelaar FDJ, Jansen MR, Lohuis SJ et al (2023) The need for routine native nephrectomy in the workup for kidney transplantation in autosomal dominant polycystic kidney disease patients. Urol Int 107(2):148–156 35810740 10.1159/000525575 PMC9945191

[40] Patel P, Horsfield C, Compton F, Taylor J, Koffman G, Olsburgh J (2011) Native nephrectomy in transplant patients with autosomal dominant polycystic kidney disease. Ann R Coll Surg Engl 93(5):391–395 21943464 10.1308/003588411X582690 PMC3365458

[41] Maxeiner A, Bichmann A, Oberländer N, El-Bandar N, Sugünes N, Ralla B et al (2019) Native nephrectomy before and after renal transplantation in patients with autosomal dominant polycystic kidney disease (ADPKD). J Clin Med 8(10):1622 31590248 10.3390/jcm8101622 PMC6832478

[42] Argyrou C, Moris D, Vernadakis S (2017) Tailoring the “perfect fit” for renal transplant recipients with end-stage polycystic kidney disease: indications and timing of native nephrectomy. In Vivo 31(3):307–312 28438856 10.21873/invivo.11060 PMC5461438

